# Observations on Distortions of the Spine; with a Few Remarks on Deformities of the Legs

**Published:** 1831

**Authors:** 


Observations on Distortions of the Spine; with a few Remarks on Deformities of the Legs. By *Lionel J. Beale*, Member of the College of Surgeons, &c. 8vo, stitched, pp. 102, 1831.

This is the second essay of Mr. Beale's on Spinal Distortions; or rather is an appendix to one which he published some little time ago. Works on this subject have been surprisingly numerous of late years, a circumstance owing in great measure, no doubt, to the good fortune which attended the publication of the late Mr. Shaw. Our profession is crowded with individuals naturally anxious for success. The competition is great, the struggle severe, the opportunities not numerous. One great road to fame and to riches is through the regions of the press, that almost omnipotent power. All are eager to write, but many are puzzled on what to exercise the pen. Some fortunate man makes a lucky hit; he is the envy of his brethren; he may make his fortune, but his success proves an ignis fatuus to others, and lures them to their doom. A discovery of this kind is like the discovery of an oyster-bank, the spot is instantly crowded with dredgers, and perhaps the discoverer himself is actually elbowed out. This is no false picture of medical life. A popular book is sure to be followed by half a dozen of a similar description pressing hard upon its heels, till the public become sickened, the market exhibits a glut, and the disease brings its remedy. We do not assert that this is the case with respect to books upon the spine; but we have lately been favoured with a sufficient number of them.

Mr. Beale appears to be a sensible man, not rash in drawing conclusions, nor too eager to admit every statement as fact. The present pamphlet is intended as a sort of feeler for a second edition of the former publication. If it meets with no criticism, good; if objections are started, they may be allowed or answered. Such is the profession of the author in his preface. The tract, as he terms it, is of popular construction: that is, tibia is called leg-bone, and there is a frontispiece lithograph of a distorted girl, with her chémise *en bas*, and a distorted skeleton beside her. Such appears to be the established mode of procedure in these cases, and though, professionally speaking, exceptions might be taken to the continuance of the practice, we are not disposed to be censorious, and shall make no further observations on the matter. It cannot be supposed that, under these circumstances, an analysis is necessary; it would be preposterous. We may select, however, some isolated passages for the information of our readers. The following sentiments on the use of instruments in cases of lateral curvature are judicious.

"In permanent lateral curvature, where all hope of cure must be abandoned, where no means can raise the spine to its proper position, steel supports become essential, not only for the ease of the individual, but to prevent the mischief from increasing. I have met with many cases where the deformity has increased, even after the age of 30, and in which steel supports would have been very beneficial. Prejudices against the use of such instruments are almost universal among medical men. I call them prejudices, because they are unfounded in reason, because they are conclusions drawn from witnessing the abuse of mechanical means, and because they are held in deference to the advice of surgeons, who have deprecated their use only because they themselves have never employed them. The late Mr. Shaw, in his earlier publications, was a great enemy to all mechanical support of the spine, but length of experience induced him to alter his opinions on this subject.

The attempts to cure spinal deformity by the sole aid of instruments, and the unfortunate results which have too often followed, has raised so great a dislike to mechanical aid, that among our most celebrated surgeons such means are entirely discarded. That many cases of lateral curvature have received no benefit from the long-continued use of steel stays and other supports I am ready to admit; but the reason has been, that no other means were employed, in combination with the use of instruments, to strengthen the muscles, and thus enable them, without artificial aid, to retain the spine in its proper position. Supports have been employed, under the notion that if the body could for a time be kept erect, it would, when growth had ceased, continue upright without assistance, and in some cases this has occurred. Some girls become awry in consequence of the rapidity of their growth, the bones are elongated, the muscles are consequently lengthened by the separation of their two extremities, and it is some time before their breadth measures in proportion to their length. In such cases, if, by any means, either the recumbent posture or the use of steel supports, the spine can be kept erect until growth ceases, and the muscles are fully developed, then all fear of lateral curvature will have ceased. But in the majority of cases where spine-supporters have been employed, the end has appeared to be gained by the mere concealment of the deformity; and when the unfortunate patient has attempted to lay aside her artificial support, she has found herself weaker than ever, and of necessity obliged to continue it all her life.

Instruments to support the spine are useful as adjuvants to the treatment, so long as the period of life affords a hope of cure, and afterwards they are useful in preventing an increase of deformity, and as conducing greatly to the comfort of the wearer. The obvious remedy, in that variety of lateral curvature of which we are speaking, is to improve the general health and strength, and more especially to invigorate the muscles of the spine. Now it must be apparent, that an instrument which will at all impede the motions of the spine, will prevent the possibility of effecting a cure of the distortion, and such is the case with all the machines invented for the removal, or, more properly speaking, the concealment of spinal deformity. There are few cases of lateral curvature which may not be cured, if properly treated before the cessation of growth. The incurable cases are those resulting from contractions within the chest, consequent on diseased lungs, and those dependent on the effects of inflammation of the cartilages and ligaments, where rigid contractions have taken place among these parts and the bones;—but these cases are few, in comparison with the large number dependent on the other causes enumerated." 51.

The chief point in the treatment of these cases is exercise; but the body, when at rest, must be supported, and, with this object, Mr. Beale had an instrument constructed by Mr. Eagland, calculated, in his opinion, to attain the ends proposed.

"This instrument supports the spine by embracing the ribs, and holding up the axillæ on a kind of crutch, the lower end of which rests on the seat, so that, while the patient is at her meals, or taking any lessons, the muscles of the back are relieved from all exertion. It slips over the dress, is adjusted in a minute and as soon removed. By this means and the recumbent position alternately during the intervals of exercise, the spine is never suffered to relax into that serpentine form which constitutes lateral curvature. This serpentine form is often apparent in the very earliest stage of lateral curvature, and the ingenious theory of Mr. Bell will not apply in all cases. Mr. Bell asserts that the primary curvature is in the loins, and that the upper curves are mere effects of the efforts of the muscles to restore the equilibrium. Mr. Bell's own illustration of the soldier standing at ease, when the spine assumes this serpentine form, strongly favours the opinion that, in some cases at least, the alternate curves are not consecutive, but primary, and of simultaneous origin. Weakly girls frequently indulge in this standing at ease; at length, by constant habit and too much adherence to study, the unnatural form becomes fixed, and, if neglected, after a time permanent. In most of these cases, a period of some years elapses before the distortion may be considered permanent. I have not met with one before the age of 20, where the spine, by extension, could not immediately be made more straight, proving the possibility, by proper means, of improving the form, if not of entirely rectifying it. In persons who have one leg shorter than the other, the spine is thrown into a serpentine form at every step, and yet individuals thus circumstanced pass through life without permanent lateral curvature. I instance these cases to shew how long a time it takes to make lateral curvature of the spine rigid and permanent, provided there be no disease in the bones, ligaments and cartilages.

The health always suffers in cases of lateral curvature primarily or secondarily, and it is surprising to witness the effect of exercise in improving it. Where any degree of perseverance in exercise is observed, the health speedily improves, and, if the diet be at the same time judicious, little or no medicine will be required. The great desideratum in these cases is perseverance, with which almost all may be benefited, but without it no method of treatment will avail. In the exercises usually adopted it is essential to fix the pelvis, in order that the body should bend at the loins and the muscles of the back be called into action, otherwise the whole trunk, with the pelvis, will move on the heads of the thigh-bones. This circumstance will also point out the absurdity of expecting benefit to result from exercise while the patient is wearing a steel support, the very object of which is to fix the spine immoveably on the pelvis, and thus prevent all possibility of exercise to the muscles of the back and loins. When a mechanical support is used, it should be in the intervals of exercise alone, to be taken off on every occasion when the exercises are resumed." 54.

The following picture of the effects of tight lacing is calculated to inspire horror in the minds of those deluded and deluding mothers, and self-immolated daughters, who offer up their vows to the savage godhead of Fashion. This potent deity, beneath the wheels of whose car more victims are crushed, than ever prostrated their bodies before the Jaggernaut in the Carnatic, is unquestionably a warm friend to the spine-doctors.

"Let us examine the action of one only of the numerous muscles connected with the chest and spine, the latissimus dorsi, which arises from the common tendon of the loins; the fleshy part of this muscle encircles the lower and back part of the chest, passes over the corner of the shoulder-blade, from which it receives a fleshy bundle; and as it passes over the ribs, it sends some tendinous slips to them: the lower fibres of this large muscle ascend, the upper ones go directly across, the flat tendon produced by the junction of these fibres forms the back part of the arm-pit, and is inserted into the arm-bone. The action of this muscle is to draw down the arm, and when either arm is fixed, it draws the spine to one side or the other, as in climbing, &c. How can this muscle act as it should do under the compression of tight stays? The same question would apply to others of the muscles of the spine and chest. The whole back is clothed with strong muscles, its cavities are crossed by many smaller ones related to the ribs and spine, and the actions of all are more or less impeded by the compression of stays. Look at the withered legs of the beggars in our streets, notoriously produced by tight bandages—tight stays produce a similar effect in a less degree, but sufficient to weaken the power of those muscles whose duty it is to maintain the natural position of the spine.

Not only does tight lacing act directly in this manner, but indirectly it operates in diminishing muscular vigour, by impeding respiration. It is well known that muscular power bears a relative proportion to the freedom of respiration, animals having the highest development of the respiratory organs, being the most powerful in muscular force. Tight stays compress the ribs together, and prevent the play of the respiratory muscles—when applied during the growth of the body, they prevent the development of the chest, and thus lay the foundation of many pectoral diseases. Much more might be said on the subject—to expect that stays will be banished from the female dress would be idle, but I think few mothers who will reflect on the evils of tight lacing in growing girls, will hesitate to defer at least to the latest moment, the vanity of forming their children of that shape which is most convenient to the dress-maker; for really the great use of stays, from all I can learn on the subject, appears to be, that they form the most suitable ground-work for the attachment of the manufactures of these artists. The female form, at least in youth, requires no artificial aid to improve it; who would think of putting stays on the Venus de Medici!" 46.

The latter portion of this little essay is dedicated to cases, and to the consideration of what has been denominated spinal irritation. Nothing new is offered, and having lately devoted many pages to the works of Mr. Tate, Mr. Teale, and Dr. Addison on this affection, we must hold ourselves excused for not re-entering upon it at present. At the same time we must again protest against a hasty manner of concluding that the spine is the source of the anomalous pains which young women suffer from the crown of the head to the extremity of the toes, simply because they complain of much uneasiness on pressure of particular portions of the spine. The fact is that the great majority of these patients will refer the sources of their sufferings to any point to which their attention is particularly directed by their medical attendants. On this we speak advisedly, and are prepared to substantiate by proof, if necessary, what we have asserted.

